# Prenatal Lipopolysaccharide Exposure Alters Hepatic Drug-Metabolizing Enzyme Expression in Mouse Offspring via Histone Modifications

**DOI:** 10.3390/toxics11010082

**Published:** 2023-01-15

**Authors:** Hanhan Zhu, Guangming Liu, Qi Chang, Mengyao Yan, Kun Yang, Yanxin Li, Yali Nie, Xiaotian Li, Shengna Han, Pei Wang, Lirong Zhang

**Affiliations:** 1 Department of Pharmacology, School of Basic Medical Sciences, Zhengzhou University, Zhengzhou 450001, China; 2 Department of Pharmacology, School of Pharmacy, Zhengzhou University, Zhengzhou 450001, China; 3 Translational Medical Center, Weifang Second People’s Hospital, The Second Affiliated Hospital of Weifang Medical University, Weifang 261041, China; 4 Henan Provincial People’s Hospital, Henan Eye Hospital, People’s Hospital of Zhengzhou University, Zhengzhou 450001, China

**Keywords:** inflammation, lipopolysaccharides, drug-metabolizing enzyme, histone modification

## Abstract

Inflammation is a major regulator of drug-metabolizing enzymes (DMEs), therefore contributing to the interindividual variability of drug effects. However, whether prenatal inflammation affects DMEs expression in offspring remains obscure. This study investigated the effects of prenatal lipopolysaccharide (LPS) exposure on hepatic expression of inflammatory-related genes, nuclear receptors, and DMEs in offspring mice. Prenatal LPS exposure on gestational day (GD) 10 led to higher expression of NF-κB, Pxr, and Cyp2b10, while lower expression of Car, Ahr, Cyp3a11, and Ugt1a1 in postnatal day (PD) 30 offspring. However, multiple doses of LPS exposure on GD10-14 resulted in higher levels of inflammatory-related genes, Cyp1a2, and Cyp2b10, and lower levels of Pxr and Cyp3a11 in PD30 offspring liver. For PD60 offspring, decreased hepatic expression of NF-κB and IL-6, and increased expression of Pxr and Cyp3a11 were seen in single-dose LPS groups, whereas opposite results were observed in the multiple-dose LPS groups. Notably, enhanced H3K4me3 levels in the PXR response elements of the *Cyp3a11* promoter were observed in the liver of PD60 offspring mice from dams treated with multiple doses of LPS during pregnancy. Overall, this study suggests that parental LPS exposure could persistently alter the hepatic expression of DMEs, and histone modifications may contribute to the long-term effects.

## 1. Introduction

Drug-metabolizing enzymes (DMEs) play a vital role in the biotransformation of endogenous and exogenous substances [1,2,3]. The expression level and activity of DMEs are closely related to drug efficacy and toxicity. However, there are significant inter-individual differences in the expression and activity of DMEs [4]. It is widely known that DMEs are transcriptionally regulated by multiple transcription factors, such as pregnane X receptor (PXR), constitutive androstane receptor (CAR), and aryl hydrocarbon receptor (AhR) [5,6,7]. Importantly, the expression of DMEs can be influenced by physiological and pathological conditions. Numerous studies have found that inflammation alters the expression of DMEs [8,9,10,11]. Lipopolysaccharide (LPS)-mediated alteration of DMEs depends on transcription factors [12]. Nuclear factor-κB (NF-κB) is a key regulator in the inflammatory response [13]. It can regulate the expression of DMEs directly through binding to the promoter region of DME genes or indirectly by regulating the expression of transcription factors, such as PXR and CAR [6,14,15].

Recent studies have confirmed that aberrant environmental factors during early life (including the fetus, infant, and child) can cause long-term changes in gene expression and influence adult-onset disease outcomes [16,17,18,19]. Notably, evidence has shown that early-life environmental factors can also have long-term impacts on DMEs expression, thus affecting drug efficacy in adulthood [5,20,21]. Maternal infection and inflammation are common complications during pregnancy. It is well demonstrated that maternal inflammation is likely associated with chronic diseases, including cardiovascular and metabolic diseases [19,22,23]. Thus, it is reasonable to assume that this population is more likely to be treated with medication. However, whether prenatal inflammation has long-term effects on the hepatic expression of DMEs in offspring remains to be elucidated elusive. This is meaningful for clinical precision medicine.

Epigenetic modifications, such as histone modifications, significantly contribute to the long-term effects associated with aberrant environmental events in early life [18,24,25]. Dynamic changes of histone modifications play key roles in the ontogenic expression of cytochrome P450 enzyme (CYP) 3A4 in the human liver and Cyp3a11 (human CYP3A4 homologous gene) in the mouse liver [26,27]. Notably, we previously revealed that epigenetic modifications, including histone H3 lysine 4 and 27 trimethylations (H3K4me3 and H3K27me3, respectively), contributed to the persistently altered expression of Cyp3a11 following PXR activation during early life in mice [20]. This evidence prompts us to hypothesize that prenatal LPS exposure can trigger epigenetic alterations, which may result in epigenetic memory and probably have a persistent effect on drug metabolism in adult.

In the current study, LPS was used as a model compound, and C57BL/6J mice were used as an animal model to study the impacts of prenatal LPS exposure on the hepatic expression of DMEs in offspring mice and the underlying mechanism. Maternal mice were intraperitoneally injected with a single or multiple dose of LPS (100 μg/kg) on gestational day (GD) 10 or GD10-14. The hepatic expression of inflammatory genes, transcription factors, and DMEs was detected in offspring mice at different ages. Moreover, Cyp3a11 was selected as a representative gene to study the underlying mechanism of histone modifications on the long-term effect of prenatal LPS exposure. This knowledge may prompt a new insight into the interindividual expression of DMEs and the mechanism of drug–drug interactions.

## 2. Materials and Methods

### 2.1. Chemicals and Reagents

LPS and all other reagents were purchased from American Sigma Company or as indicated in the specified methods.

### 2.2. Animals and Procedures

C57BL/6J mice (SPF grade, 6–8 weeks) were purchased from Beijing Vital River Laboratory Animal Technology Co. (Beijing, China). All mice were allowed free access to food and water and maintained on a 12-h light/12-h dark cycle at a controlled temperature (22 °C–26 °C) and humidity (40–70%) environment. Experimental procedures were carried out in accordance with institutional animal care guidelines. The animal study protocol was approved by the Animal Care Committee of Zhengzhou University. 

**Figure 1:**
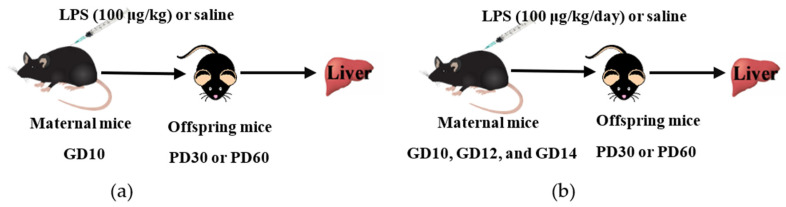
Schematic diagram of experimental design. (**a**) Impact of a single dose of LPS exposure during pregnancy on the hepatic gene expression in offspring mice at different ages. (**b**) Impact of multiple doses of LPS exposure during pregnancy on the hepatic gene expression in offspring mice at different ages. GD, gestation day; PD, postnatal day.

For mating purposes, four females were housed overnight with two males starting at 9:00 p.m. The detection of a vaginal plug the following morning was as an index of gestation and designated as the gestational day (GD)0. The day of birth was considered as postnatal day (PD)0. To study the effect of a single-dose LPS exposure during pregnancy on the offspring mice, dams were intraperitoneally (i.p.) injected with LPS (100 μg/kg) or saline (vehicle) on GD10, as described in Figure 1a. To investigate the effect of multiple-dose LPS exposure during pregnancy on the offspring mice, dams were administered LPS (100 μg/kg, i.p.) or saline on GD10, GD12, and GD14, as shown in Figure 1b. The single-dose LPS study and the multiple-dose LPS study were performed separately. Offspring mice were sacrificed on PD30 or PD60 (equivalent to adolescent and adult age, respectively). Liver tissues were collected, frozen rapidly in liquid nitrogen, and stored at −80 °C for further analysis. 

### 2.3. Total RNA Isolation

Total RNA was isolated from approximately 50 mg of frozen liver tissue using the TriPure isolation reagent (Roche Company, Basel, Switzerland) following the manufacturer’s instructions. The concentration of total RNA was quantified spectrophotometrically at 260 nm, and the purity was confirmed by 260/280 using a NanoDrop One instrument (Thermo Scientific Company, Waltham, MA, USA). cDNAs were synthesized from total RNA using the PrimeScript RT Reagent kit (TaRaKa Biotechnology, Dalian, China) according to the manufacturer’s protocol and stored at −20 °C.

### 2.4. Quantitative Real-Time Polymerase Chain Reaction

Quantitative real-time polymerase chain reaction (qRT-PCR) was performed using an SYBR Premix EX Taq kit (TaRaKa Biotechnology, Dalian, China) with specific primers (Supplemental Table S1) in the QuantStudio5 fluorescent quantitative PCR instrument (Thermo Scientific Company). The expression level of GAPDH was used as a reference. The relative mRNA expression level of each gene was calculated according to the 2^-ΔΔCT^ method. 

### 2.5. Western Blot Analysis 

Whole protein homogenates of mouse liver samples were prepared using a total protein-extraction reagent for mammalian tissues (Boster Bio, Wuhan, China) with a protease inhibitor cocktail reagent (Roche Company, Basel, Switzerland). A bicinchoninic acid method was used to determine the protein concentration following the manufacturer’s instructions (Beyotime Institute of Biotechnology, Hangzhou, China). 

The protein extract (100 μg/mL) was separated on a 10% SDS-PAGE gel using the Bio-Rad electrophoresis system and electro-blotted onto a PVDF membrane (0.45 μm) using the Bio-Rad transfer system (Bio-Rad Company, Hercules, CA, USA). The membranes were blocked with 5% non-fat milk at room temperature for 1.5 h and then incubated with a primary antibody against CYP3A11 (MAB10041, 1:1000, EMD Millipore Corporation) at 4 °C overnight. A primary antibody against GAPDH (6004-1-Ig, 1:10,000, Proteintech, Wuhan, China) was used as a loading control. Immunoreactive bands were detected by chemiluminescence using corresponding horseradish peroxidase-conjugated secondary antibodies (SA00001-1, 1:10,000, Proteintech). The ECL chemiluminescence method was used to detect the target protein band in the FlourChenTM instrument (Protein Simple Company, Minneapolis, MN, USA), and the protein band of GAPDH was used as the internal reference. Protein Intensities bands were quantified using the ImageJ software (National Institutes of Health, Bethesda, MD, USA).

### 2.6. Chromatin Immunoprecipitation Analysis

Chromatin Immunoprecipitation (ChIP) analysis was performed using frozen liver samples, as described previously [20,28,29], with minor modifications. Briefly, approximately 100 mg liver samples were crosslinked with 1% formaldehyde for 10 min and halted by 125 mM glycine. Samples were then subjected to lysis using an SDS lysis buffer (1% SDS, 50 mM Tris-HCl (pH 8.0), 2 mM EDTA, 0.1% sodium deoxycholate, and 1% Triton-X) with a proteinase inhibitor cocktail on ice for 30 min. The lysates were sonicated with a Bioruptor Pico sonication system (Diagenode, Liège, Belgium) to shear DNAs into 200 to 1000-bp size. The sonication condition was 30 s on and 30 s off for 40 cycles. The fragment size was confirmed by agarose gel electrophoresis. ChIP-grade antibodies against H3K4me3 (9751S, Cell Signaling Technology, Boston, MA, USA) and H3K27me3 (9733S, Cell Signaling Technology) were used for immunoprecipitation. A mouse IgG antibody (12-371B, EMD Millipore) was used as a negative control. The immunoprecipitation and DNA purification procedures were described clearly in the previous study [29]. The purified DNA was determined by qRT-PCR with specific primers (Supplemental Table S2) using an SYBR Premix EX Taq kit (Takara Bio) in the QuantStudio5 fluorescent quantitative PCR instrument (Thermo Scientific Company).

### 2.7. Statistical Analysis

Data are expressed as mean ± SD. All statistical analyses were performed using the SPSS software version 20.0 (IBM Company, New York, NY, USA). Statistical significance between the two groups was analyzed by unpaired Student’s *t*-test. *p* < 0.05 was considered statistically significant. 

## 3. Results

To investigate the impact of maternal LPS exposure on drug metabolism in offspring throughout postnatal maturation, a mouse model was used. As shown in Figure 1, a single or multiple dose of LPS was administered to maternal mice at GD10 or GD10-14. The mRNA expression of interleukin 6 (IL-6), tumor necrosis factor α (TNF-α), transcription factors (including NF-κB, Pxr, Car, and Ahr), and DMEs (including Cyp3a11, Cyp1a2, Cyp2b10, and Ugt1a1) was detected in the liver of offspring mice at different ages. 

CYP3A4, the main phase I enzyme in the human liver, is responsible for the biotransformation of approximately 50% of the clinically used drugs [2,30]. Moreover, significant inter-individual expression of CYP3A4 is well documented. CYP3A4 is responsible for individual differences in drug response and drug-drug interaction. Given the crucial role of CYP3A4 in human clinical drug metabolism, Cyp3a11 (human CYP3A4 homologous gene) was selected as a representative in the current study. Therefore, the protein expression of Cyp3a11 and the role of histone modifications in the long-term alteration of Cyp3a11 were demonstrated. 

### 3.1. Prenatal LPS Exposure Alters the Hepatic Expression of Inflammatory-Related Genes in Offspring Mice at Different Ages

**Figure 2:**
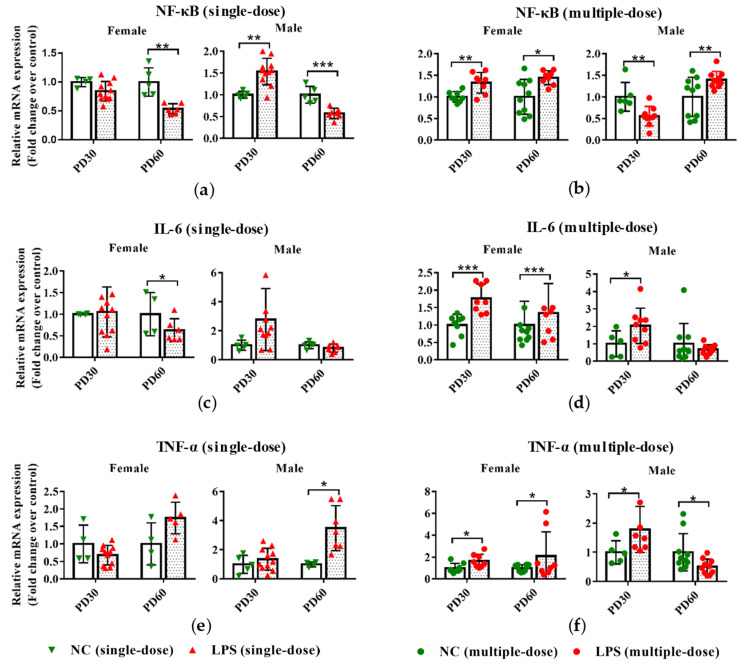
Impact of prenatal LPS exposure on the hepatic expression of inflammatory genes in offspring mice. Pregnant mice were injected with a single dose of LPS on GD10 (**a**,**c**,**e**) or a multiple dose of LPS on GD10-14 (**b**,**d**,**f**). Offspring mice were sacrificed on PD30 or PD60 to determine the hepatic gene expression. qRT-PCR analysis was conducted to detect the mRNA expression of NF-κB, IL-6, and TNF-α in the liver of offspring mice (n = 4–11/group). Values are presented as mean ± SD. * *p* < 0.05, ** *p* < 0.01, *** *p* < 0.001 vs. the gender- and age-related control group.

As seen in Figure 2a, a single dose of LPS exposure during pregnancy led to increased NF-κB expression in the liver PD30 male offspring mice and decreased mRNA expression of NF-κB in PD60 offspring mice. However, prenatal exposure to multiple doses of LPS resulted in higher mRNA expression of NF-κB in PD30 female and PD60 offspring mice and lower of it in PD30 male offspring (Figure 2b). 

For IL-6, compared with the gender and age-related control groups, its expression was lower in the PD60 female offspring of maternal mice exposed to a single-dose LPS (Figure 2c). However, in PD30 offspring and PD60 female offspring whose mothers were exposed to LPS on GD10-14, the expression of IL-6 was higher than that in the control groups (Figure 2d). 

Compared with the gender and age-related control groups, maternal exposure to a single-dose LPS led to the upregulated hepatic expression of TNF-α in PD60 male offspring (Figure 2e). Conversely, maternal exposure to multiple doses of LPS resulted in downregulated hepatic expression of TNF-α in PD60 male offspring (Figure 2f). In the liver of PD30 offspring, the TNF-α expression in mRNA level was also increased in the multiple-dose LPS-treated groups compared with the control groups (Figure 2f).

These results indicated that single-dose or multiple-dose LPS exposure during pregnancy affected the expression levels of inflammation-related genes (NF-κB, IL-6, and TNF-α) in the livers of offspring mice. 

### 3.2. Prenatal LPS Exposure Affects the Hepatic Expression of Transcription Factors in Offspring Mice at Different Ages 

**Figure 3:**
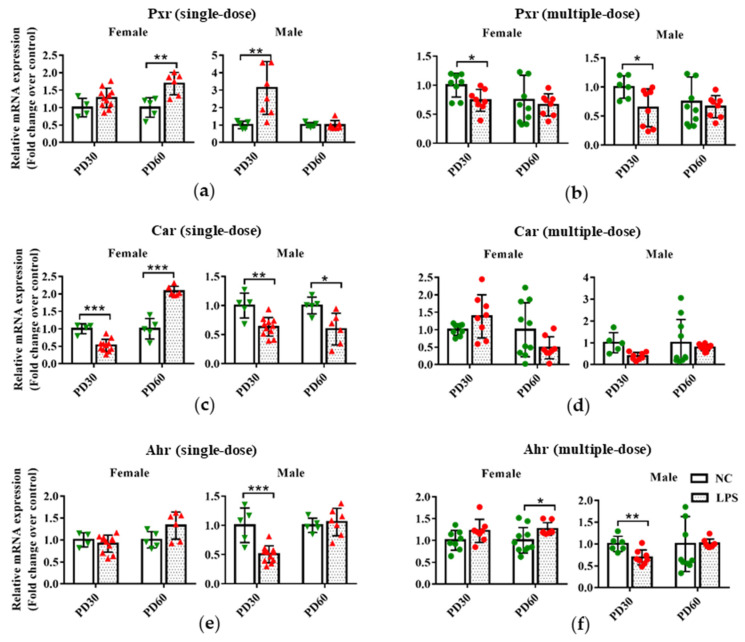
Impact of prenatal exposure to LPS on the hepatic expression of transcription factors in offspring mice. Pregnant mice were injected with a single dose of LPS on GD10 (**a**,**c**,**e**) or a multiple dose of LPS on GD10-14 (**b**,**d**,**f**). Offspring mice were sacrificed on PD30 or PD60 to detect gene expression. qRT-PCR analysis was conducted to detect the mRNA expression of Pxr, Car, and Ahr in the liver of offspring mice (n = 4–11/group). Values are presented as mean ± SD. * *p* < 0.05, ** *p* < 0.01, *** *p* < 0.001 vs. the gender and age-related.

As shown in Figure 3a,b, compared with the gender and age-related control groups, PD60 female and PD30 male offspring mice in the single-dose LPS groups had a higher hepatic expression of Pxr in mRNA level, but PD30 female and PD60 male offspring mice in the multiple-dose LPS groups had lower hepatic Pxr expression.

In the single-dose LPS exposure groups, the hepatic mRNA expression of Car significantly decreased in PD30 male and female offspring and PD60 female offspring but increased in the liver of PD60 male offspring mice, compared with the gender and age-related control groups (Figure 3c). There was no statistical difference in the mRNA expression of Car between the multiple-dose LPS exposure group and the related control group (Figure 3d).

For Ahr, either a single or multiple dose of LPS exposure during pregnancy resulted in a lower expression in the liver of PD30 male offspring compared with the related control groups (Figure 3e,f). However, higher hepatic expression of Ahr was observed in PD60 female offspring of the multiple-dose LPS exposure group than that in the age and gender-related control groups (Figure 3f).

These findings indicate that maternal exposure to a single or multiple dose of LPS during pregnancy affected the expression levels of hepatic transcription factors (Pxr, Car, and Ahr) in offspring mice. 

### 3.3. Prenatal LPS Exposure Impacts the Hepatic Expression of DMEs in Offspring Mice at Different Ages 

**Figure 4:**
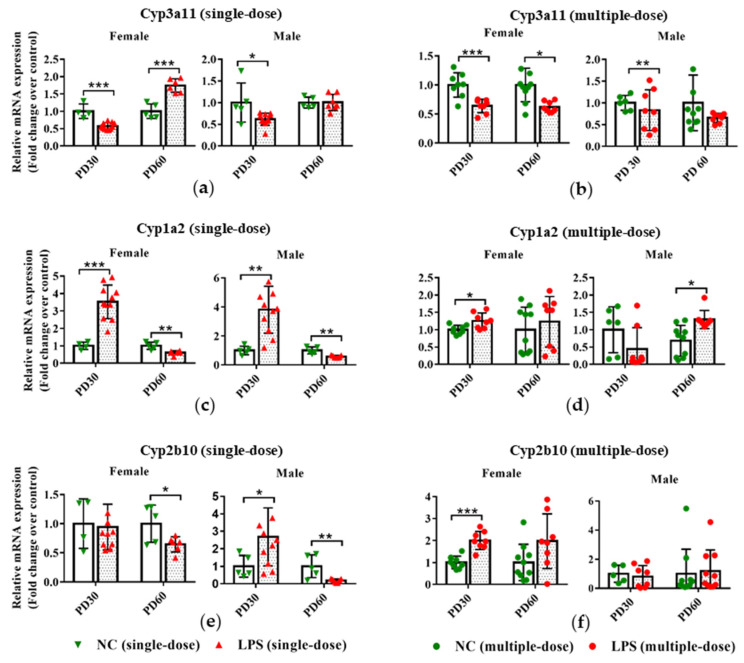

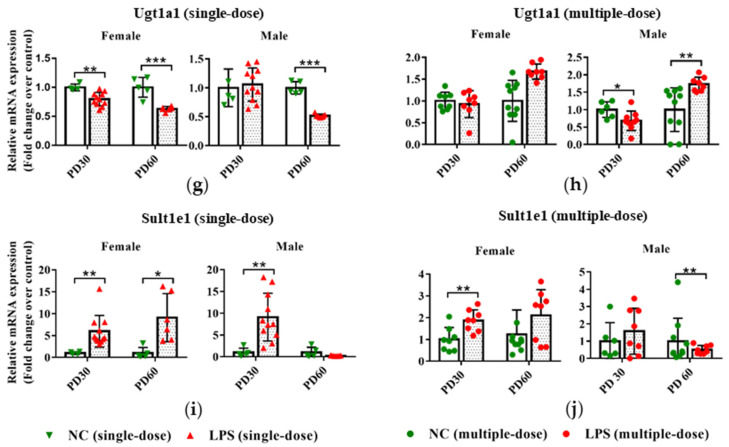
Impact of prenatal exposure to LPS on the hepatic expression of DMEs in offspring mice. Pregnant mice were injected with a single dose of LPS on GD10 (**a**,**c**,**e**,**g**,**i**) or multiple doses of LPS on GD10-14 (**b**,**d**,**f**,**h**,**j**). Offspring mice were sacrificed on PD30 or PD60 to detect gene expression. qRT-PCR analysis was conducted to detect the mRNA expression of Cyp3a11, Cyp1a2, Cyp2b10, Ugt1a1, and Sult1e1 in the liver of offspring mice (n = 4–11/group). Values are presented as mean ± SD. * *p* < 0.05, ** *p* < 0.01, *** *p* < 0.001 vs. the gender and age-related control group.

As depicted in Figure 4a,b, prenatal exposure to either a single-dose or multiple-dose LPS led to decreased mRNA expression of Cyp3a11 in the liver of PD30 female offspring. For PD60 offspring, compared with the gender and age-related control group, higher mRNA expression of Cyp3a11 was observed in female and not in male offspring delivered by single-dose LPS-treated maternal mice. Significantly lower mRNA expression of Cyp3a11 was seen in PD60 female offspring of multiple-dose LPS groups.

In the single-dose LPS exposure groups, the mRNA expression of Cyp1a2 was elevated in the PD30 offspring but reduced in the PD60 offspring, compared with the gender and age-related control groups (Figure 4c). Prenatal exposure to a multiple dose of LPS increased the hepatic mRNA expression levels of Cyp1a2 in PD30 female and PD60 male offspring (Figure 4d). Similarly, the mRNA expression of Cyp2b10 was also decreased in the PD60 offspring of the single-dose LPS exposure group but increased in the PD30 male offspring of the single-dose LPS exposure group and in the PD30 female offspring of the multiple-dose LPS exposure group (Figure 4e,f).

As shown in Figure 4g,i, prenatal exposure to a single dose of LPS tended to decrease the Ugt1a1 expression in PD30 female and PD60 offspring but increase the Sult1e1 expression in mRNA levels in the liver of PD30 and PD60 female offspring. In the multiple-dose groups, the mRNA expression of Ugt1a1 was altered in male offspring due to prenatal LPS exposure (Figure 4h). Higher mRNA expression of Sult1e1 in the PD30 female offspring and lower expression of it in the PD60 male offspring were observed, compared with the gender and age-related control groups (Figure 4j).

**Figure 5:**
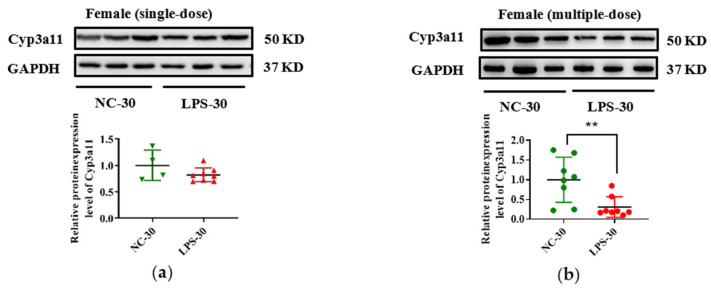

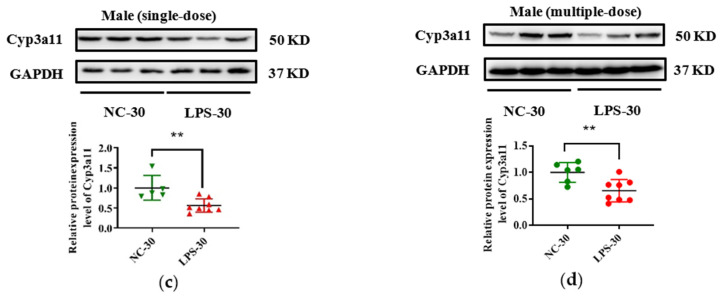
Prenatal exposure to LPS altered the protein expression of Cyp3a11 in the liver of PD30 offspring mice. Pregnant mice were injected with a single dose of LPS on GD10 (**a**,**c**) or multiple doses of LPS on GD10-14 (**b**,**d**). The hepatic protein expression of Cyp3a11 in offspring mice at day 30 after birth was determined by Western blot assay (n = 5–8/group). ** *p* < 0.01 vs. NC-30. NC-30, 30-day-old offspring mice of the control group; LPS-30, 30-day-old offspring mice of the LPS-treated group.

**Figure 6:**
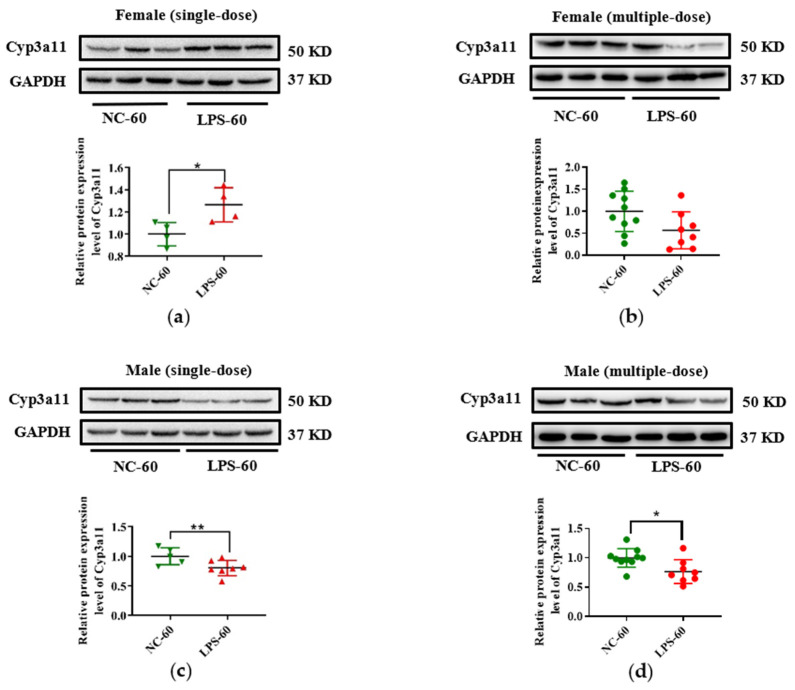
Prenatal exposure to LPS altered the protein expression of Cyp3a11 in the liver of PD60 offspring mice. Pregnant mice were injected with a single dose of LPS on GD10 (**a**,**c**) or multiple doses of LPS on GD10-14 (**b**,**d**). The hepatic protein expression of Cyp3a11 in offspring mice at day 60 after birth was determined by Western blot assay (n = 5–10/group). * *p* < 0.05, ** *p* < 0.01 vs. NC-60. PD60, postnatal day 60; NC-60, 60-day-old offspring mice of the control group; LPS-60, 60-day-old offspring mice of LPS treated group.

In addition, due to the vital role of human CYP3A4 in drug metabolism, Cyp3a11 was selected as a representative gene, and its expression in protein level was measured in this study. As seen in Figure 5, consistent with the decrease of Cyp3a11 in mRNA levels in LPS exposure groups, maternal exposure to either a single or multiple dose of LPS during pregnancy resulted in reduced protein expression of Cyp3a11 in the liver of PD30 offspring. For PD60 offspring mice, the alterations of Cyp3a11 expression in protein level in the single-dose LPS groups were consistent with that in mRNA level (Figure 6a,c). Moreover, in the multiple-dose LPS groups, the protein expression levels of Cyp3a11 were also significantly decreased (Figure 6b,d). 

### 3.4. Histone Modifications Contribute to the Decreased Cyp3a11 Expression Following Prenatal Exposure to Multiple Doses of LPS 

Growing evidence has demonstrated that alterations of histone modifications by environmental exposure during early life can result in persistent changes in gene expression in later life [24,31]. Therefore, the role of histone modifications (H3K4me3 and H3K27me3) in the decreased expression of Cyp3a11 following prenatal LPS exposure was investigated in the present study.

**Figure 7:**
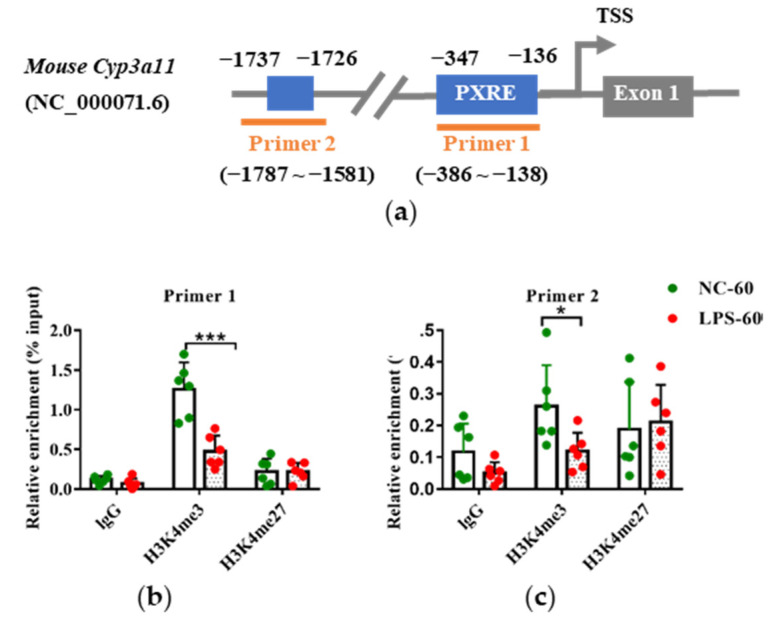
Prenatal exposure to multiple doses of LPS altered the histone modification levels in the *Cyp3a11* promoter in the PD60 female offspring mouse liver. Pregnant mice were injected with multiple doses of LPS on GD10-14. The liver samples of PD60 female offspring were collected and subjected to ChIP analysis (n = 6). (**a**) Schematic locations of the PXR response elements (PXREs) and ChIP-qPCR primers in the *Cyp3a11* promoter. (**b**) H3K4me3 enrichment fold around the PXREs in the *Cyp3a11* promoter. (**c**) H3K27me3 enrichment fold around the PXREs in the *Cyp3a11* promoter. Values are presented as mean ± SD. * *p* < 0.05, *** *p* < 0.001 vs. control group. NC-60, 60-day-old offspring mice of the control group; LPS-60, 60-day-old offspring mice of LPS treated group.

The enrichment levels of H3K4me3 and H3K27me3 around the PXR response elements (PXREs) in the promoter of *Cyp3a11* were detected. ChIP analysis was conducted in six randomly selected liver samples of PD60 female offspring which maternal mice were treated with vehicle or LPS on GD10-14 (Figure 1b). ChIP-qPCR primers were designed to cover the two PXREs (−347 to −136 and −1737 to −1726, respectively) in the *Cyp3a11* promoter (Figure 7a). Consistent with the decreased expression of Cyp3a11, LPS exposure groups appeared to reduce the levels of H3K4me3, an active epigenetic mark, in the two PXREs regions of *Cyp3a11* (Figure 7b). Though the enriched levels of H3K27me3, a gene silencing mark, tended to increase in the LPS exposure groups, there was no statistical significance between the LPS group and the control group (Figure 7c). These findings indicate that histone modifications (H3K4me3) may contribute to the persistent downregulation of Cyp3a11 by prenatal LPS exposure. 

## 4. Discussion

Accumulating evidence indicates that aberrant environmental events during development can lead to epigenetic alterations in the genome that permanently influence health in later life [24,31]. In this study, we demonstrate that LPS exposure during early life results in a selective/persistent alteration of DMEs expression. Notably, we reveal that decreased H3K4me3 enrichment levels in the *Cyp3a11* promoter are an underlying mechanism for the persistently decreased expression of Cyp3a11 in offspring mice due to prenatal exposure to multiple doses of LPS.

Abundant evidence has highlighted that pregnancy is a critical window of developmental plasticity [32,33]. For prenatal inflammation exposure-programmed diseases, such as hypertension, diabetes, and heart disease, the second trimester of gestation is the most sensitive time window [19,34,35]. In the current study, the exposure time of LPS was also selected in the second trimester of gestation. The endpoint ages of offspring mice were chosen to mimic the stages of adolescent and adult, based on the ontogenic expression pattern of DMEs [36,37]. Two recent studies show that prenatal LPS exposure induces transgenerational inheritance of hypertension [19,38]. Whether prenatal inflammation has a longer-term effect on hepatic DMEs expression and drug metabolism should be investigated in further research.

In previous studies, to investigate the effects of maternal LPS exposure on PXR and Cyp3a11 expression in placental and fetal mouse liver, ICR mice were injected intraperitoneally with LPS (0.1–0.5 mg/kg) on GD17 [39,40,41]. Twelve hours after LPS treatment, Xu and colleagues found that the mRNA expression of Pxr and Cyp3a11 in the placenta and fetal liver decreased whilst upregulating heme oxygenase-1 in the fetal liver [39,40,41]. Here, we observed differential impacts of prenatal exposure to a single or multiple dose of LPS on offspring at different ages. For instance, following maternal exposure to a single-dose LPS, the mRNA and protein expression of Cyp3a11 was significantly reduced in the liver of PD30 female offspring mice, but in PD60 offspring was induced. However, following a multiple-dose LPS exposure during pregnancy, Cyp3a11 expression was significantly downregulated in PD30 and PD60 female offspring mice, which is consistent with the literature. The differential results may be attributed to the terminal point of the study and the mouse species. There is evidence that a single low dose of LPS stimulation can significantly increase the expression of Pxr and its target gene Cyp3a11 in adult rats, which may be a compensatory mechanism, while repeated low dose of LPS stimulation can significantly decrease the expression of Pxr and its target gene Cyp3a11 in the nucleus [42].

NF-κB is a curial regulator of inflammatory cytokines. LPS exposure during pregnancy has been shown to significantly increase the expression of NF-κB and other inflammatory factors in the fetal liver [43]. AhR, a ligand-activated transcription factor, mainly regulates the expression of CYP1A. Recent studies have shown that AhR is indispensable for the LPS-induced inflammatory response [12,44,45]. During inflammation, the activation of NF-κB can upregulate the expression of AhR. In the present study, it was found that exposure to a single or multiple dose of LPS during pregnancy altered the mRNA expression of NF-κB in the liver of PD30 and PD60 offspring mice. Consistently, in the multiple doses of LPS groups, the expression of NF-κB and Ahr was both increased in the PD60 female offspring and decreased in the PD30 male offspring. Previous studies have shown that Pxr can regulate the basal expression of DMEs such as Cyp3a11 and Cyp2b10. Therefore, we speculate that exposure to LPS during pregnancy can increase the mRNA expression level of NF-κB in the fetal liver, affect the expression of liver nuclear receptor genes in the liver, and then interfere with the expression and development of DMEs regulated by it. This effect will continue to affect the expression of DMEs in mice from birth to adulthood. The involvement of NF-κB in the long-term alteration of DMEs following prenatal LPS treatment needs to be investigated in the future.

Gender differences in DMEs expression in response to LPS treatment were observed in the current study. We found that prenatal exposure to multiple-dose LPS led to significantly induced expression of Cyp1a2, Cyp2b10, Sult1e1 in PD30 offspring females but not in males. This phenomenon may be due to the sexual dimorphism of DMEs expression at basal levels. Sexual difference induction of DMEs expression is commonly observed in mice [46,47]. This may be due to the sexual dimorphism of DMEs expression at the basal level.

Studies examining the molecular mechanism of early developmental exposure to adverse environmental factors have focused on epigenetic markers (such as DNA methylation and histone modification) [16]. Evidence shows that exposure to LPS during pregnancy can downregulate the mRNA expression levels of Pxr and Cyp3a11 in the placenta and fetal liver of female mice. We previously illustrated that H3K4me2/3 and H3K27me3 contributed to both the ontogenic expression of CYP3A4 in human livers and the PXR-mediated induction of CYP3A4 by rifampicin in LS174T cells [26,29]. Consistent with the elevated Cyp3a11 expression, lower H3K4me3 enrichment levels in the *Cyp3a11* promoter were observed in the liver of PD60 offspring of multiple doses of LPS treatment groups. Presumably, maternal exposure to multiple doses of LPS perturbs the histone modifications and results in a persistent alteration of epigenetic memory. The deeper molecular mechanisms have not been explored in the current study. Based on our previous study [29], the recruitment of histone methyltransferase-related factors NCOA6 and p300 to the *Cyp3a11* promoter by PXR may be responsible for the persistent alterations of H3K4me3 and H3K27me3 in the *Cyp3a11* promoter, which will be investigated in the future. Future studies will also be performed to investigate the H3K4me3 and H3K27me3 levels in promoters of other DMEs. Notably, recent studies show that N6-methyl-adenosine (m6A) modification contributes to adverse pregnancy outcomes [48,49]. Moreover, m6A modification is involved in the regulation of DMEs [50,51]. m6A modifying proteins showed ontogenic changes in mRNA levels along with liver development [52,53]. The role of m6A in the long-term altered Cyp3a11 expression in offspring due to prenatal inflammation is a new research direction.

In the current study, only wild-type C57BL/6J mice were utilized to investigate the long-term effects of prenatal LPS exposure on the expression of DMEs in the offspring. However, it is well documented that species differences exist in the expression and activation profiles of DMEs between humans and mice [54]. To better extrapolate the present finding to humans, it is important to perform studies using a humanized mouse model and to assess the enzyme activity of DMEs. Whether the metabolism of clinically used drug is affected by prenatal LPS exposure also needs to be addressed in further study.

## 5. Conclusions

In summary, this study showed that prenatal exposure to either a single dose or multiple dose of LPS has long-term impacts on the hepatic expression of DMEs in offspring. Notably, the results indicate that decreased H3K4me3 enrichment levels in the promoter of *Cyp3a11* may contribute to reduced expression of Cyp3a11 following prenatal LPS stimulus. These findings promote an understanding of the long-term effect of inflammation during pregnancy on drug metabolism in offspring.

## Data Availability

The authors confirm that the data supporting the findings of this study are available within the article.

## Supplementary Materials

The following supporting information can be downloaded at: https://www.mdpi.com/article/10.3390/toxics11010082/s1, Table S1. Primers for RT-qPCR. Table S2. Primer Sequences for ChIP-qPCR.
