# Supplementary material for: Prenatal Lipopolysaccharide Exposure Alters Hepatic Drug-Metabolizing Enzyme Expression in Mouse Offspring via Histone Modifications
Source: Toxics. 2023 Jan 15;11(1):82. doi: 10.3390/toxics11010082 (PMC9866336; doi:10.3390/toxics11010082)
Supplement: Supplementary file 1 [file toxics-11-00082-s001.zip › toxics-2126843-supplementary.pdf]

Supplemental Table S1. Primers for RT-qPCR

Gene		Sequence 5'-3'	Gene ID	Position
NF-κB	Forward	ATGGCAGACGA TGA TCCCTAC	NM0086	503-613
	Reverse	TGTTGACAGTGGTATTTCTGGTG	89.2	
IL-6	Forward	TAGTCCTTCCTACCCCAATTTCC	NM0311	566-641
	Reverse	TTGGTCCTTAGCCACTCCTTC	68.2	
TNF-α	Forward	CCCTCACACTCAGATCATCTTCT	NM0136	397-457
	Reverse	GCTACGACGTGGGCTACAG	93.3	
Pxr	Forward	CCCATCAACGAGAGGAGGA	NM0109	408-524
	Reverse	TCTGAAAAACCCCTTGCATC	36.3	
Car	Forward	GCCTAAGAGAAACAGGAGACCAT	NM0012	504-579
	Reverse	CTCCCAGCAAACGGACAGAT	43062.1	
Ahr	Forward	ACA TACGCCGGTAGGAAGAGA	NM0013	603-1343
	Reverse	GGTCCAGCTCTGTA TTGAGGC	14027.1	
Cyp3a11	Forward	ACAAACAAGCAGGGA TGGAC	NM0078	65-148
	Reverse	GGTAGAGGAGCACCAAGCTG	18.3	
Cyp1a2	Forward	GACA TGGCCTAACGTGCAG	NM0099	1005-1137
	Reverse	GGTCAGAAAGCCGTGGTTG	93.3	
Cyp2b10	Forward	AAGGAGAAGTCCAACCAGCA	NM0099	847-993
	Reverse	CTCTGCAACA TGGGGGTACT	99.4	
Ugt1a1	Forward	GTCA TCCAAAGACTCGGGCA	NM2016	1134-1294
	Reverse	GACA TTCAGGGTCACCCCAG	45.2	
Sult1e1	Forward	ATGGAGACTTCTA TGCCTGAGT	NM0231	153-284
	Reverse	ACACAACTTCACTAA TCCAGGTG	35.2	
Gapdh	Forward	AGGTCGGTGTGAACGGA TTTG	NM0012	100-222
	Reverse	TGTAGACCATGTAGTTGAGGTCA	89726.1	

Supplemental Table S2. Primer Sequences for ChIP-qPCR

Primer	Sequence (5’-3’)
Primer 1	F: CCTGCTTCTACTGTCTGAGTGT
	R: TCAAGCCAGTAGATGGATCACC
Primer 2	F: TGGTCTCTTGGATCCTGGTTTC
	R: GCACCACATACCCACCAAGA

F, forward primer; R, reverse prim
